# COVID-19 vaccination timing, relative to acute COVID-19, and subsequent risk of long COVID

**DOI:** 10.1016/j.ebiom.2026.106382

**Published:** 2026-07-24

**Authors:** Zachary Butzin-Dozier, Yunwen Ji, Lin-Chiun Wang, A. Jerrod Anzalone, Jeremy Coyle, Rachael V. Phillips, Rena C. Patel, Jing Sun, Eric Hurwitz, Sarang Deshpande, Junming (Seraphina) Shi, Andrew Mertens, Mark J. van der Laan, John M. Colford, Alan E. Hubbard, National Clinical Cohort Collaborative (N3C) Consortium, Adam B. Wilcox, Adam M. Lee, Alexis Graves, Alfred (Jerrod) Anzalone, Amin Manna, Amit Saha, Amy Olex, Andrea Zhou, Andrew E. Williams, Andrew Southerland, Andrew T. Girvin, Anita Walden, Anjali A. Sharathkumar, Benjamin Amor, Benjamin Bates, Brian Hendricks, Brijesh Patel, Caleb Alexander, Carolyn Bramante, Cavin Ward-Caviness, Charisse Madlock-Brown, Christine Suver, Christopher Chute, Christopher Dillon, Chunlei Wu, Clare Schmitt, Cliff Takemoto, Dan Housman, Davera Gabriel, David A. Eichmann, Diego Mazzotti, Don Brown, Eilis Boudreau, Elaine Hill, Elizabeth Zampino, Emily Carlson Marti, Emily R. Pfaff, Evan French, Farrukh M. Koraishy, Federico Mariona, Fred Prior, George Sokos, Greg Martin, Harold Lehmann, Heidi Spratt, Hemalkumar Mehta, Hongfang Liu, Hythem Sidky, J.W. Awori Hayanga, Jami Pincavitch, Jaylyn Clark, Jeremy Richard Harper, Jessica Islam, Jin Ge, Joel Gagnier, Joel H. Saltz, Joel Saltz, Johanna Loomba, John Buse, Jomol Mathew, Joni L. Rutter, Julie A. McMurry, Justin Guinney, Justin Starren, Karen Crowley, Katie Rebecca Bradwell, Kellie M. Walters, Ken Wilkins, Kenneth R. Gersing, Kenrick Dwain Cato, Kimberly Murray, Kristin Kostka, Lavance Northington, Lee Allan Pyles, Leonie Misquitta, Lesley Cottrell, Lili Portilla, Mariam Deacy, Mark M. Bissell, Marshall Clark, Mary Emmett, Mary Morrison Saltz, Matvey B. Palchuk, Melissa A. Haendel, Meredith Adams, Meredith Temple-O’Connor, Michael G. Kurilla, Michele Morris, Nabeel Qureshi, Nasia Safdar, Nicole Garbarini, Noha Sharafeldin, Ofer Sadan, Patricia A. Francis, Penny Wung Burgoon, Peter Robinson, Philip R.O. Payne, Rafael Fuentes, Randeep Jawa, Rebecca Erwin-Cohen, Rena Patel, Richard A. Moffitt, Richard L. Zhu, Rishi Kamaleswaran, Robert Hurley, Robert T. Miller, Saiju Pyarajan, Sam G. Michael, Samuel Bozzette, Sandeep Mallipattu, Satyanarayana Vedula, Scott Chapman, Shawn T. O’Neil, Soko Setoguchi, Stephanie S. Hong, Steve Johnson, Tellen D. Bennett, Tiffany Callahan, Umit Topaloglu, Usman Sheikh, Valery Gordon, Vignesh Subbian, Warren A. Kibbe, Wenndy Hernandez, Will Beasley, Will Cooper, William Hillegass, Xiaohan Tanner Zhang

**Affiliations:** a Stanford University School of Medicine, Stanford, CA, USA; b School of Public Health, University of California, Berkeley, Berkeley, CA, USA; c University of Nebraska Medical Center, Omaha, NE, USA; d University of Alabama at Birmingham, Birmingham, AL, USA; e Bloomberg School of Public Health, Johns Hopkins University, Baltimore, MD, USA; f University of North Carolina at Chapel Hill, Chapel Hill, NC, USA

**Keywords:** COVID-19, Long COVID, Post-acute sequelae of COVID-19, Vaccination

## Abstract

**Background:**

Vaccination is a vital tool in preventing acute COVID-19 and may confer additional protection against Long COVID, although it is unclear whether this protection wanes over time.

**Methods:**

We assessed electronic health record (EHR) data from a national, retrospective cohort of patients, comparing the 12-month cumulative incidence of Long COVID (ICD-10 code U09.9) among (A) patients who were vaccinated versus unvaccinated (two or more versus zero doses) and (B) patients diagnosed with acute COVID-19 1–3 months, 3–5 months, or 5–7 months after vaccination.

**Findings:**

In our binary cohort (*n* = 519,980), we found that patients who were vaccinated had a lower risk of Long COVID (adjusted risk ratio 0.84 (0.81, 0.88)) or mortality (adjusted risk ratio 0.83 (0.81, 0.86)) than patients who were unvaccinated. In our longitudinal cohort (*n* = 1,085,291), we did not find significant heterogeneity in Long COVID risk during the seven months following vaccination.

**Interpretation:**

We found that COVID-19 vaccination was protective against Long COVID, and we did not observe a significant waning of this protection within seven months after vaccination.

**Funding:**

This research was financially supported by the 10.13039/100000060National Institute of Allergy and Infectious Diseases (1K01AI182501 to Zachary Butzin-Dozier) and a Global Development grant (OPP1165144) from the 10.13039/100000865Bill & Melinda Gates Foundation to the 10.13039/100005595University of California, Berkeley, CA, USA. Individual authors were supported by the following funding sources: 10.13039/100000025NIMHR01131542 (PI Rena C. Patel), Jerrod Anzalone is supported by the 10.13039/100000057National Institute of General Medical Sciences, U54 GM115458, which funds the Great Plains IDeA-CTR Network. The content is solely the responsibility of the authors and does not necessarily represent the official views of the NIH.

**Research in context**

**Evidence before this study**

Long COVID, or post-acute sequelae of COVID-19, is a debilitating condition with significant long-term health impacts, yet effective preventive strategies remain limited. Vaccination against COVID-19 is critical for preventing severe COVID-19, but its role in mitigating the risk of Long COVID, particularly with respect to vaccination timing relative to infection, is not well established.

**Added value of this study**

Using longitudinal targeted maximum likelihood estimation applied to large-scale electronic health record (EHR) data from the National Clinical Cohort Collaborative (N3C), encompassing over one million patients, our study provides robust evidence on how vaccination timing influences Long COVID risk. We found vaccinated individuals had a significantly lower risk of developing Long COVID compared to unvaccinated individuals (adjusted marginal risk ratio: 0.84, 95% CI: 0.81, 0.88). Furthermore, we found no significant waning of vaccine protection against Long COVID within seven months post-vaccination.

**Implications of all the available evidence**

This study supports and extends prior evidence regarding the protective effect of COVID-19 vaccination against Long COVID, emphasising that vaccine-induced protection does not significantly diminish within seven months after vaccination. These findings provide critical evidence to inform public health strategies, clinical recommendations, and vaccination policies aimed at minimising the long-term burden of COVID-19.

## Introduction

Researchers have made major steps toward understanding, preventing, and treating acute COVID-19, but Long COVID remains a debilitating and poorly understood condition. Five years have passed since the COVID-19 pandemic began, and many individuals have grown fatigued with repeated vaccination booster doses, particularly given the common perception of reduced severity of acute infection since the Omicron wave began in late 2021.^1^, ^2^, ^3^

Systematic reviews have noted a lack of randomised trials evaluating the relationship between COVID-19 vaccination and Long COVID, and have observed considerable heterogeneity in the observational findings of studies addressing this research question.^4^^,^^5^ Brannock et al. found that COVID vaccination was protective against the subsequent risk of Long COVID using a large, nationally sampled US cohort (adjusted odds ratio 0.66, 95% CI (0.55, 0.80)).^6^ Although recent studies have found a protective effect of COVID vaccination on the risk of Long COVID,^6^, ^7^, ^8^, ^9^, ^10^ less is known about the relationship between the timing of vaccination and additional doses relative to acute COVID-19 and the development of Long COVID. Vaccination timing is important in designing and promoting effective clinical guidance for COVID-19 vaccination schedules and recommendations.

We sought to evaluate the hypothesis that a longer time interval between the most recent vaccination and COVID-19 infection would be associated with a higher risk of Long COVID diagnosis. These findings may be relevant for policymakers, researchers, and clinicians. An improved understanding of the relationship between vaccination timing and Long COVID can inform policymakers’ guidelines regarding booster vaccination schedules, and these results can also be used by public health officials to motivate individuals to follow these recommendations.

## Methods

Few studies have evaluated the relationship between vaccination timing and long-term sequelae of acute COVID, including Long COVID. Two significant barriers to this research question are (1) methodological challenges to causal inference in observational settings with longitudinal confounding, high dimensionality, and a large proportion of non-random missingness, and (2) the lack of available longitudinal or biomarker data with sufficient sample size. These challenges can be overcome, respectively, through a targeted machine learning (i.e., causal inference) approach to analysing data from the National Clinical Cohort Collaborative (N3C).

N3C is one of the largest publicly available sources of electronic health record data, encompassing records for over 22 million patients from 84 healthcare institutions.^11^ Investigators have leveraged N3C data to produce hundreds of publications in collaboration with experts nationwide.^12^ This national sample provides an excellent opportunity for investigators to conduct research using high-dimensional, harmonised data from electronic health records.

We estimated our target causal parameter of interest while adjusting for relevant covariates and accounting for heterogeneous monitoring. We applied Longitudinal Targeted Maximum Likelihood Estimation to generate a marginal structural model (LTMLE, R package “ltmle”).^13^, ^14^, ^15^, ^16^, ^17^ LTMLE enables the estimation of causal parameters (in this case, hazard ratios) from nonparametric measures of association that are interpretable to non-statisticians (e.g., clinicians, policymakers), and the estimators used were doubly robust and maximally efficient. We estimated the parameters of interest using the LTMLE package and used generalised linear models as candidate algorithms to estimate the components of the longitudinal data-generating distribution. In our binary cohort, we applied Targeted Maximum Likelihood Estimation^18^^,^^19^ and defined binary exposure (yes/no) over the entire period of risk (see Binary cohort). See Supplemental Material 1 for details on our causal parameters of interest and relevant assumptions.

### Shared methods across longitudinal and binary cohorts

#### Exclusion criteria

As both documentation of COVID-19 vaccination and Long COVID diagnosis are highly heterogeneous by study site, we only included study sites that report at least 5% of their patients have at least one COVID-19 vaccination or booster dose (approximately 2/3 the CDC-reported vaccination rate over the study period, which is consistent with previous studies)^6^ and report that at least 1% of their patients have been diagnosed with Long COVID (consistent with previous studies)^6^^,^^20^^,^^21^ at some point during the N3C observation window (2018 to present) to prevent differential misclassification of the exposure or outcome of interest. We excluded patients with a Long COVID diagnosis within the 4 weeks after the index COVID-19 infection date (as most definitions of Long COVID require that approximately one month must pass after acute COVID-19 for a patient to be considered “at risk” for Long COVID).^21^, ^22^, ^23^, ^24^

#### Covariates

We included covariates via the N3C Logic Liaison Fact Table (data dictionary previously published; see Supplemental Material 1)^25^ that were associated with and plausibly caused (i.e., temporally preceded) the exposure and outcome (i.e., assessed at baseline). Patient characteristics included sex (assigned sex at birth), age (years), race, ethnicity, body mass index, immunocompromised status (based on documented diagnoses), systemic corticosteroid use (any prescription before inclusion), depression, chronic lung disease, hypertension, obesity, diabetes, tobacco use (whether the patients was ever a tobacco smoker before inclusion), asthma, date of inclusion (index vaccination date or acute COVID-19 date), and healthcare utilisation rate (healthcare interactions per month; healthcare utilisation is highly associated with Long COVID diagnosis).^26^, ^27^, ^28^ We adjusted for data partner (i.e., study site) and source common data model format (i.e., data documentation format) to account for geospatial trends and systematic variations in health documentation formats.^26^ In other words, we sought to adjust for systematic differences between contributing data sites based on heterogeneous (1) viral transmission characteristics and (2) data documentation.

#### COVID-19 and long COVID

We defined acute COVID-19 based on either a laboratory diagnostic positive result (either PCR or antigen) or a provider diagnosis (ICD-10-CM U07.1).^29^ If both indications were present, we included the earliest date as the index infection date. Our primary outcome of interest was the cumulative incidence of Long COVID 1–12 months after acute COVID-19. We defined Long COVID as the diagnosis of post-acute sequelae of COVID-19 (PASC, ICD-10 code U09.9).^22^^,^^23^

### Binary cohort: vaccinated versus unvaccinated

We evaluated binary vaccination status (unvaccinated versus vaccinated) before acute COVID-19. We hypothesised that COVID-19 vaccination before acute COVID-19 would be protective against Long COVID.

#### Inclusion criteria

We included participants with documented COVID-19 between December 25, 2021 (CDC-reported data of Omicron variant dominance in the US)^30^ and September 25, 2022.^29^

#### Exposure of interest

We defined vaccination as a binary variable indicating whether the patient received at least two doses of a COVID-19 vaccination (or booster) before their index acute COVID-19 date. We excluded patients who only received one vaccination dose before acute COVID-19 to avoid including those who were partially vaccinated (i.e., incomplete vaccination), which is consistent with previous studies.^6^

### Longitudinal cohort: vaccination timing relative to acute COVID-19

We hypothesised that a greater time between the most recent vaccination and COVID-19 is associated with a greater risk of Long COVID diagnosis.

#### Inclusion criteria, sample size, and power

We included participants with a documented COVID-19 vaccine or booster dose between December 25, 2021 (CDC-reported data of Omicron variant dominance in the US)^30^ and September 25, 2022 (to allow for sufficient follow-up time) (see Supplemental Material 2 for details on study timeline).

#### Exposures

The primary exposure of interest was the time (months) between the initial vaccination or booster date and the date of acute COVID-19. We operationalised this exposure by defining “baseline” (start of follow-up) for each individual as the first COVID-19 vaccination or booster received between December 25, 2021, and September 25, 2022.^30^ We evaluated these patients’ acute COVID-19 status between 1 and 7 months following the vaccination date. We limited observation to this 6-month interval to account for the frequent missingness of vaccination data in N3C. By only including patients with a documented vaccination, at the time of vaccination, and observing subsequent vaccination and acute COVID-19 status for 7 months, we have created a cohort where we are confident in patient vaccination status during this observation period (as few patients received multiple vaccination doses during this observation period). During this interval, we also evaluated additional vaccination doses and healthcare utilisation rate, as healthcare utilisation is strongly associated with Long COVID diagnosis.^21^^,^^26^ If the patient was diagnosed with acute COVID-19 during this period, we monitored their Long COVID status in the 12 months following acute COVID-19. We partitioned time in two-month intervals, where each patient has a binary status for COVID-19 vaccination/booster, healthcare utilisation (i.e., at least one healthcare interaction), and Long COVID diagnosis in each two-month interval (one to three months [1≤ *t* < 3], three to five months [3 ≤ *t* < 5], or five to seven [5 ≤ *t* < 7] months). Patients who were diagnosed with COVID-19 more than 7 months after vaccination, or were not diagnosed with COVID-19 after vaccination during our observation period, remained in our analytic sample and contributed to overall model fit although they were not included in one of these three treatment groups.

#### Analysis methods

We applied Longitudinal Targeted Maximum Likelihood Estimation (LTMLE) to assess the causal impact of vaccination timing relative to COVID-19 on the risk of Long COVID.^13^, ^14^, ^15^, ^16^ These risk ratios reflect the relationship between time since vaccination and the risk of Long COVID.

#### Secondary analyses

Susceptibility to symptomatic COVID-19 soon after COVID-19 vaccination may indicate an underlying predisposition or risk for COVID-19, due to an immunocompromising condition. As a sensitivity analysis, we conducted a subgroup analysis of “low-risk” individuals to evaluate whether we observed similar temporal relationships between COVID-19 vaccination timing relative to acute COVID-19 and subsequent Long COVID risk. This “low-risk” subgroup was defined by: age at acute COVID-19 between 18 and 65 years, no chronic lung disease, and no immunocompromised status. As definitions of time periods of Long COVID susceptibility vary, we conducted sensitivity analyses in both our binary and longitudinal cohorts, where we defined Long COVID as a diagnosis taking place between 3 and 12 months after acute COVID-19. As a sensitivity analysis for our definition of exposure in our binary cohort (2 vaccination doses versus zero vaccination doses), we compared the risk of Long COVID between patients who had received 2 or more doses versus 1 or fewer vaccination doses. We sought to explore the relationship between vaccination and severe post-COVID outcomes by evaluating the relationship between vaccination status and 12-month mortality risk following acute COVID-19.

#### Ethics approval and consent to participate

This study was approved by the UC Berkeley Office for Protection of Human Subjects (2022-01-14980). The N3C data transfer to NCATS is performed under a Johns Hopkins University Reliance Protocol # IRB00249128 or individual site agreements with NIH. N3C received a waiver of consent from the NIH Institutional Review board and allows the secondary analysis of these data without additional consent.

#### Role of funders

The Funders had no role in study design, data analyses, interpretation, or manuscript preparation.

## Results

Our binary cohort of vaccinated (2 or more doses) versus unvaccinated (0 doses) patients included 519,980 patients, where 55% were vaccinated before acute COVID-19 (Table 1). Generally, patients who were vaccinated had higher healthcare utilisation (0.7 versus 0.5 visits per month), a greater comorbidity burden (29% versus 20% systemic corticosteroid use, 13% versus 10% lung disease, 13% versus 8% diabetic, 5% versus 3% immunocompromised, and 28% versus 19% hypertensive) and were older (16% versus 9% over 70 years old) than patients who were unvaccinated.

**Table 1:** Characteristics of sample patients.

Panel A. Patient characteristics in the longitudinal cohort. Grouped by months from most recent SARS-CoV-2 vaccination to infection. Data not shown: Patients who were not diagnosed with COVID-19 during follow-up.						Panel B. Patient characteristics in the binary cohort. Grouped by SARS-CoV-2 vaccination status (2 or more vaccination doses versus no vaccination doses).	
	Value	1–3 months, *n* (%)	3–5 months, *n* (%)	5–7 months, *n* (%)	7+ months, *n* (%)	Vaccinated, *n* (%)	Unvaccinated, *n* (%)
Total		23,619 (16%)	32,047 (21%)	33,820 (22%)	62,111 (41%)	288,359 (55%)	231,621 (45%)
Sex	Male	9782 (41%)	13,101 (41%)	13,761 (41%)	25,464 (41%)	120,324 (42%)	96,666 (42%)
Age	(0.0, 17.0]	1316 (6%)	1609 (5%)	1832 (5%)	3016 (5%)	Excluded	Excluded
	(17.0, 49.0]	4857 (21%)	9293 (29%)	10,778 (32%)	17,648 (28%)	145,162 (50%)	149,687 (65%)
	(49.0, 70.0]	8168 (35%)	10,320 (32%)	11,526 (34%)	21,313 (34%)	97,336 (34%)	61,133 (26%)
	(70.0, 107.0]	9276 (39%)	10,823 (34%)	9683 (29%)	20,132 (32%)	45,861 (16%)	20,801 (9%)
Race and ethnicity	White Non-Hispanic	13,432 (57%)	18,048 (55%)	19,081 (55%)	34,837 (55%)	192,331 (67%)	154,398 (67%)
	Black or African American Non-Hispanic	3394 (14%)	4695 (15%)	4966 (15%)	9373 (15%)	37,650 (13%)	30,099 (13%)
	Hispanic or Latino Any Race	3892 (16%)	5431 (17%)	5644 (17%)	10,206 (16%)	29,865 (10%)	24,140 (10%)
	Other Non-Hispanic	415 (2%)	560 (2%)	579 (2%)	1087 (2%)	11,772 (4%)	9489 (4%)
	Unknown	1206 (5%)	1642 (5%)	1750 (5%)	3237 (5%)	9982 (4%)	7948 (3%)
	Asian or Pacific Islander Non-Hispanic	1126 (5%)	1468 (5%)	1577 (5%)	2936 (5%)	5080 (2%)	4170 (2%)
Body mass index	[0.0, 25.0)	3309 (14%)	4316 (14%)	4317 (13%)	7844 (13%)	28,172 (10%)	19,087 (8%)
	[25.0, 30.0)	5080 (22%)	6356 (20%)	6253 (18%)	12,453 (20%)	47,979 (17%)	26,607 (12%)
	[30.0, 35.0)	10,848 (46%)	14,904 (46%)	15,776 (47%)	27,661 (45%)	158,960 (55%)	151,967 (66%)
	[35.0, 40.0)	2179 (9%)	3221 (10%)	3605 (11%)	6810 (11%)	25,787 (9%)	15,667 (7%)
	[40.0, 100.0)	2194 (9%)	3245 (10%)	3851 (11%)	7326 (12%)	27,242 (9%)	18,141 (8%)
Medical conditions	Systemic corticosteroid use	9992 (42%)	13,013 (41%)	13,261 (39%)	26,515 (43%)	83,879 (29%)	45,878 (20%)
	Lung disease	5093 (22%)	6736 (21%)	7216 (21%)	14,466 (23%)	37,130 (13%)	23,493 (10%)
	Diabetes	4598 (20%)	6038 (19%)	6588 (20%)	12,841 (21%)	36,884 (13%)	19,649 (8%)
	Other immunocompromised	2686 (11%)	3268 (10%)	3198 (10%)	6301 (10%)	15,642 (5%)	7055 (3%)
	Smoking	1657 (7%)	2562 (8%)	2961 (9%)	5882 (10%)	18,355 (6%)	20,121 (9%)
	Hypertension	10,529 (45%)	13,471 (42%)	13,757 (41%)	27,443 (45%)	80,737 (28%)	43,074 (19%)
	Depression	5035 (21%)	7093 (22%)	7627 (23%)	14,789 (24%)	41,467 (14%)	24,652 (11%)
Medical utilisation	Visits per month, Mean (SD)	1.55 (1.63)	1.5 (1.67)	1.45 (1.57)	1.57 (1.7)	0.72 (0.86)	0.47 (0.62)
	Number of previous vaccinations, mean (SD)	3.33 (0.97)	3.17 (0.97)	3 (0.98)	3.05 (0.96)	2.57 (0.68)	0 (0)

Our longitudinal cohort included data from 1,085,291 patients who were vaccinated against COVID-19 between December 2021 and September 2022 (not mutually exclusive). Of the patients who were diagnosed with COVID-19 after vaccination during follow-up, 16% were diagnosed with acute COVID-19 one to three months after vaccination, 21% were diagnosed with acute COVID-19 three to five months after vaccination, 22% were diagnosed with acute COVID-19 5 to 7 months after vaccination, and 41% were diagnosed with acute COVID-19 7+ months after vaccination. Patients across groups had similar healthcare utilisation rates to one another (1.5 to 1.6 interactions per month). Patients who were vaccinated one to three months before acute COVID-19 had the highest number of previous COVID-19 vaccinations (3.3), followed by 3-5 months (3.2), 7+ months (3.05), and 5-7 months (3.0). Patient age, body mass index, and comorbidity burden were similar across groups.

### Binary cohort

**Fig. 1:**
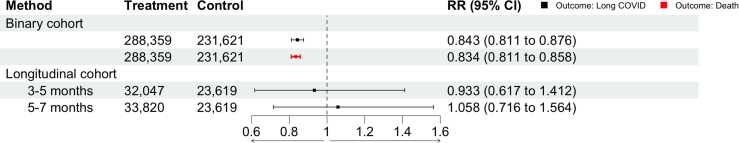
Relationship between vaccination status and 12-month cumulative incidence of Long COVID (black) or mortality (red). In the binary cohort (*n* = 519,980), “treatment” refers to patients with 2 or more COVID-19 vaccination doses, while “control” refers to patients with no COVID-19 vaccination doses. In the longitudinal cohort (*n* = 1,085,291), “control” refers to patients who were vaccinated 1–3 months before acute COVID-19, while “treatment” refers to patients who were vaccinated either 3–5 months or 5–7 months before acute COVID-19, respectively. The centre point represents the risk ratio point estimate, and error bars represent 95% confidence intervals. RR: Risk ratio.

In our adjusted model, we found that patients who were vaccinated against SARS-CoV-2 before acute COVID-19 had a lower risk of subsequent Long COVID, compared to patients with no documented SARS-CoV-2 vaccination before acute COVID-19 (RR 0.843, 95% CI (0.811, 0.876)), over the study period (Fig. 1). We observed a similar relationship between SARS-CoV-2 vaccination and Long COVID risk after restricting to Long COVID diagnoses between 3 and 12 months after acute COVID-19 (RR 0.827, 95% CI (0.738, 0.926); Supplemental Material 3). After evaluating our secondary outcome of mortality, we found that SARS-CoV-2 vaccination was associated with a lower risk of mortality in the 12 months following acute COVID-19 (risk ratio 0.834, 95% CI (0.811, 0.858)) (Supplemental Material 4). In a sensitivity analysis comparing patients with 2 or more vaccination doses to 1 or fewer vaccination doses, our observed relationship shifted towards the null hypothesis (risk ratio 0.978, 95% CI (0.944, 1.015)) (Supplemental Material 4).

### Longitudinal cohort (vaccination timing and long COVID)

We did not find a significant relationship between COVID-19 vaccination timing, relative to acute COVID-19, and subsequent risk of Long COVID in the first 7 months following vaccination. Compared to patients who experienced acute COVID-19 1–3 months after vaccination/booster, patients who experienced COVID-19 3–5 months after vaccination/booster had a Long COVID risk ratio of 0.933 (0.617, 1.412), and patients who experienced COVID-19 5–7 months after vaccination/booster had a Long COVID risk ratio of 1.058 (0.716, 1.564). The marginal adjusted, 12-month risk of Long COVID among patients who experienced acute COVID-19 one to three months after their most recent COVID-19 vaccination or booster was 0.005, for patients who experienced COVID-19 three to five months after vaccination/booster was 0.005, and for patients who experienced COVID-19 5–7 months after vaccination/booster was 0.006. We observed a similar relationship among low-risk patients (18–65 years old, not immunocompromised, and no lung disease), and we observed a similar relationship after restricting to Long COVID diagnoses between 3 and 12 months after acute COVID-19 (Supplemental Material 3, Supplemental Material 5). We found that patients who were vaccinated more recently, relative to acute COVID-19, were more likely to receive a SARS-CoV-2 booster in the 12 months after acute COVID-19 (Supplemental Material 6).

## Discussion

We found that COVID-19 vaccination before acute COVID-19 was protective against subsequent Long COVID. We did not find evidence of waning protection of SARS-CoV-2 immunisation concerning Long COVID in patients who experienced COVID-19 between 1 and 7 months following vaccination. Our finding that pre-COVID-19 vaccination against SARS-CoV-2 is associated with 0.83 times the risk of mortality in the 12 months after acute COVID-19 further underscores the ability of vaccination to limit long-term negative outcomes of acute COVID-19. These findings build on our understanding of COVID-19 vaccination effectiveness, particularly regarding Long COVID.

Given the current context of COVID-19 transmission and complex exposure profiles, our findings contrast the potential impacts of vaccine-induced immunity versus infection-induced immunity on long-term health outcomes. Our study found that SARS-CoV-2 vaccination is associated with a lower risk of long-term consequences of COVID-19, such as Long COVID or mortality, which is consistent with previous studies.^4^, ^5^, ^6^^,^^31^^,^^32^ Our point estimate regarding the association between COVID-19 vaccination and subsequent Long COVID risk is more modest than some previous evaluations (risk ratio of 0.84 versus odds ratio of 0.66)^6^ and is reflective of the heterogeneity in the documentation of both COVID-19 vaccination and Long COVID in electronic health records. In contrast to SARS-CoV-2 vaccination, recent studies have consistently found that SARS-CoV-2 reinfection is associated with increased risk of Long COVID and related long-term consequences (e.g., heart disease), compared to a single SARS-CoV-2 infection.^33^^,^^34^ These cumulative findings are consistent in demonstrating that SARS-CoV-2 vaccination before acute COVID-19 may be protective against long-term consequences of COVID-19, while repeated SARS-CoV-2 infection may increase the risk of these consequences.

Our findings regarding the relationship between vaccination timing and Long COVID are consistent with a previous retrospective cohort study that found that SARS-CoV-2 vaccination effectively protects against Long COVID for at least 6 months.^35^ Previous studies have shown that vaccination effectiveness against acute COVID-19 wanes over time.^36^^,^^37^ On the other hand, immunisation against diseases like smallpox can lead to long-term protection even after circulating antibodies wane.^38^ Further research is needed to evaluate whether the effectiveness of SARS-CoV-2 vaccination, with respect to Long COVID, wanes over time.

Systematic reviews have noted a lack of randomised trials evaluating this relationship and have noted considerable heterogeneity in observational evaluations of this relationship, which supports the need for additional research in this area.^4^^,^^5^ In these settings, a causal inference (Targeted Machine Learning) approach can provide crucial evidence to approximate causal parameters via observational data.^13^, ^14^, ^15^^,^^19^^,^^39^^,^^40^ The approach described here provides a replicable method for evaluating the impact of post-acute COVID-19 vaccination timing on subsequent Long COVID risk, as several studies have hypothesised that SARS-CoV-2 vaccination after acute COVID-19 may be protective against Long COVID.^4^^,^^41^ Furthermore, these methods can be replicated across a range of time-dependent, pre-exposure (e.g., pre-exposure prophylaxis), post-exposure (e.g., post-exposure prophylaxis), and sequential interventions to determine optimal intervention timing to minimise subsequent outcomes.

### Limitations

Due to the high variability of our findings (i.e., wide confidence intervals) in the longitudinal cohort, we do not interpret our results as a “true null” (i.e., there may be a relationship between vaccination timing and Long COVID). In other words, we do not conclude that the effectiveness of COVID-19 vaccination, with respect to Long COVID, does not wane over time. A study with greater power may detect a statistically significant relationship between vaccination timing and Long COVID. Future studies should further evaluate the relationship between vaccination timing and Long COVID through a data source with (1) higher quality vaccination data, (2) greater sensitivity of Long COVID outcome assessment, (3) a longer period of follow-up between vaccination and acute COVID-19, and (4) more complete patient health information (i.e., lower risk of bias due to differential healthcare utilisation). In our longitudinal cohort, we addressed limitations in vaccination data quality (i.e., high missingness) by defining baseline as the date of a patient’s vaccination and limiting the exposure observation period to 7 months.

As is common with data from electronic health records (EHR), N3C has incomplete reporting of patient outcomes. This includes covariates related to baseline health and comorbidities, vaccination status, acute COVID-19 diagnosis, and Long COVID diagnosis. Previous studies using N3C have addressed these concerns.^6^^,^^42^^,^^43^ Notably, patient health information skews toward patients with high medical utilisation rates, severe illness, or hospitalisation.^42^ Therefore, our approach sought to characterise and account for this missingness, while transparently addressing limitations to generalisability. We did not evaluate effect modification by vaccination type (e.g., Pfizer versus Johnson & Johnson) due to the high proportion of missingness of this information. One key method, in addition to longitudinal multivariate adjustment and weighting, is that we restricted study sites to only include data providers that reported at least 5% of their patients had at least one COVID-19 vaccination or booster dose and reported that at least 1% of their patients have been diagnosed with Long COVID at some point during the N3C observation window.

### Strengths

A major contribution of this study is the analytic method we applied. This study incorporated Longitudinal Targeted Maximum Likelihood Estimation to determine the relationship between high-dimensional covariates, multiple time-dependent exposures (vaccination and acute COVID-19), and longitudinal outcomes while intervening on heterogeneous monitoring. No single regression approach could characterise the exposure-outcome relationship due to time-dependent confounding. These methods can be applied to a range of research questions, particularly questions related to vaccination timing.

The data source, N3C, is another strength of this study. Given the potentially subtle association between vaccination timing and Long COVID, a large data source is needed to detect possible relationships. Furthermore, without randomisation, high-dimensional covariate data are needed to adjust for imbalance. N3C provides this large and high-dimensional data source, which is necessary to address this research question.

### Conclusions

COVID-19 vaccination appears protective against Long COVID, and we did not find evidence that this protection wanes within 7 months of vaccination. This contributes to the body of literature supporting COVID-19 vaccination as a crucial public health measure.

## Contributors

All authors read and approved the final version of the manuscript.

Authorship was determined using ICMJE recommendations.

ZB: Generated research question, drafted manuscript, managed project timeline, and coordinated analysis.

YJ, LW, JA, JC, RVP, RCP, JS, SD, EH, JS, AM, ML, JC, and AH: Provided oversight on study design and analysis plan, supported analysis, reviewed manuscript, and provided feedback.

ZB, YJ, and LW have accessed and verified the underlying data.

Consortial contributors: clinical data model expertise; data curation; data integration; data quality assurance; funding acquisition; governance; critical revision of the manuscript for important intellectual content; N3C Phenotype definition; project evaluation; project management; regulatory oversight/admin (see Supplemental Material 7).

## Data sharing statement

All analytic code and data are available in the N3C Enclave by request. Access to the N3C Data Enclave is managed by NCATS (https://ncats.nih.gov/research/research-activities/n3c/resources/data-access). Interested researchers must first complete a data use agreement, and next a data use request, in order to access the N3C Data Enclave. Once access is granted, the N3C data use committee must review and approve all use of data and the publication committee must approve all publications involving N3C data.

## Declaration of interests

*Competing interests*: The authors declare no competing interests.
