# Supplementary material for: COVID-19 vaccination timing, relative to acute COVID-19, and subsequent risk of long COVID
Source: eBioMedicine. 2026 Jul 24;130:106382. doi: 10.1016/j.ebiom.2026.106382 (PMC13445364; doi:10.1016/j.ebiom.2026.106382)
Supplement: Supplementary file 1 [file mmc1.pdf]

Supplemental Material 1

August 29, 2025

1 Longitudinal analysis

We observe n independent and identically distributed samples of

$$O = \big(L(0), C(1), L(1), A(1), Y(1), \dots, C(K), A(K), Y(K)\big) \sim P_0,$$

where each $k \in \{1, \dots, K-1\}$ corresponds to a two-month interval, up to $k = 6$. The baseline covariates $L(0)$ include age, sex, and other characteristics related to COVID and Long COVID incidence. Missing baseline values were imputed using the median, with an additional indicator variable denoting imputation. For $k \geq 1$, $L(k)$ contains time-varying covariates, including vaccination and booster history and a visit indicator.

The treatment variables $A(k)$ represent the occurrence of COVID-19 infection at time k, and the censoring indicator $C(k)$ equals 1 if death was observed at time k. The outcome $Y(k)$ is the incidence of long COVID, assessed at each time point k.

The final time point $K = 7$ defines a variable-length follow-up interval. The length of this interval depends on the timing of COVID-19 infection, ensuring that all patients contribute an equal 12-month outcome period, rather than allowing those infected earlier to contribute disproportionately longer follow-up. Further details on the construction of this outcome period are provided in Supplemental Material 2.

Unlike $A(1), \dots, A(6)$, the variable $A(7)$ does not denote COVID-19 infection. Instead, it indicates whether long COVID status was measured during the follow-up ($A(7) = 1$ if measured; $A(7) = 0$ otherwise). This distinction is important because our analysis assumes an intervention that eliminates loss to follow-up among participants without long COVID up to $k = 6$. Such an intervention guarantees ascertainment of long COVID status for all eligible participants.

1.1 Identification

Following the notations commonly used in the LTMLE literature Lendle et al. 2017, we denote by an overbar the history of a variable up to a given time. For

example, $\bar{L}(k)$ represents $(L(0), L(1), \ldots, L(k))$. We consider a static treatment regime $\bar{a}^{\text{treat}} = (a_k : k \in \{0, \ldots, K\})$.

Our causal parameter of interest is the risk ratio comparing a treatment regime $\bar{a}^{\text{treat}}$ with a reference regime $\bar{a}^{\text{ref}}$:

$$RR = \frac{E_{P_0}\big(Y^{\bar{a}^{\text{treat}}}\big)}{E_{P_0}\big(Y^{\bar{a}^{\text{ref}}}\big)}.$$

Here, $\bar{a}^{\text{ref}} = (1, 0, \ldots, 1)$ corresponds to COVID incidence in the first 1–3 months after baseline, along with a recorded visit during the follow-up period. Alternative regimes may represent, for instance, incidence in months 3–5, $\bar{a}^{\text{treat}} = (0, 1, 0, \ldots, 1)$, or in months 5–7, $\bar{a}^{\text{treat}} = (0, 0, 1, \ldots, 1)$. The counterfactual outcome $Y^{\bar{a}^{\text{treat}}}$ denotes the long COVID status that would be observed if a subject had followed exposure regime $\bar{a}^{\text{treat}}$, same for $\bar{a}^{\text{ref}}$.

Let $d \in \mathcal{D}$ denote a treatment regime from the set of regimes of interest. To identify the causal parameter, we require the following assumptions (Petersen et al. 2014):

- **Sequential randomization (no unmeasured confounding):** $Y^d \perp A_k \mid pa(A_k)$ for $k = 1, \ldots, K$. This requires that, at each time k, all common causes of L and $A(k)$ are observed and included. While we believe our dataset captures strong predictors of long COVID, some unmeasured confounding may remain.
- **Positivity:** $P_0\big(A(k) = d_k \mid \bar{L}(k), \bar{A}(k-1) = d_{k-1}\big) > 0$ almost everywhere. In practice, each subject must have a nonzero probability of following the regime of interest at every time point, conditional on past history. This holds in our setting, given that the treatment regimes involve patients acquiring COVID after vaccination.

Under these assumptions, the counterfactual means $E_{P_0}\big(Y^{\bar{a}^{\text{treat}}}\big)$ and $E_{P_0}\big(Y^{\bar{a}^{\text{ref}}}\big)$ can be identified via the longitudinal G-computation formula (Robins 1986).

1.2 Estimation

For any time point t, define the regression of $Y(t)$ on observed past covariates:

$$\begin{aligned} \bar{Q}^{a,t}_{L(k)} &= E_0\big(Y(t) \mid \bar{L}(k-1), \bar{A}(k-1) = a_{k-1}\big) \\ &= E_0\Big(\bar{Q}^{a,t}_{L(k+1)} \mid \bar{L}(k-1), \bar{A}(k-1) = a_{k-1}\Big). \end{aligned} \tag{1}$$

Thus, the counterfactual mean $E_{P_0}\big(Y^a\big)$ can be expressed as $\bar{Q}^{a,t}_{L(0)}$, defined recursively as a sequence of conditional expectations (Bang and Robins 2005).

We implemented estimation using the `ltmle` package. This approach relies on estimates of the sequential regressions $\bar{Q}^{a,t}_{L(k)}$ and of the intervention mechanism components $g_{1,k}$ and $g_{2,k}$ (Lendle et al. 2017). In our analysis, both the sequential regressions $\bar{Q}$ and the intervention mechanism g were estimated with generalized linear models.

2 Binary analysis

We observe n i.i.d. copies of O with data structure $O = (W, A, \Delta, \Delta Y) \sim P_0 \in \mathcal{M}$, where $W \in \mathbb{R}^p$ denotes baseline covariates, $A \in \{0, 1\}$ is the indicator of complete vaccination, and $\Delta Y \in \{0, 1\}$ is observed Long COVID diagnosis. Δ refers to patient observation during the outcome period.

Our target parameter is

$$\frac{EY^1}{EY^0},$$

where Y^a is the counterfactual under $A = a$. Under standard positivity and no unmeasured confounding, the target parameter can be identified by:

$$E_{P_0}\big(E_{P_0}(Y \mid A = 1, W, \Delta = 1) - E_{P_0}(Y \mid A = 0, W, \Delta = 1)\big).$$

3 Variable definitions

In this analysis, we used covariates created in the N3C Logic Liaison Table (Zhou 2025), which aims to provide harmonized data for use across replicable resource projects. The Logic Liaison Data Dictionary describes all included data in detail (Zhou 2025). Below, we describe how our features correspond with the variable names in the data dictionary, which we indexed at time periods described above.

We calculated healthcare utilization as the number of patient healthcare interactions (`total_visits`) per month over the observation period (`observation_period`).

Number of previous vaccinations at baseline: `total_number_of_COVID_vaccine_doses`

Race and ethnicity: `race_ethnicity`

sex: `sex`

age (years): `age`

body mass index: `BMI_max_observed_or_calculated`

immunocompromised status (based on documented diagnoses): `OTHERIMMUNOCOMPROMISED_indicator`

systemic corticosteroid use (any prescription before inclusion): `SYSTEMICCORTICOSTEROIDS_indicator`

depression: `DEPRESSION_indicator`

chronic lung disease: `CHRONICLUNGDISEASE_indicator`

hypertension: `HYPERTENSION_indicator`

obesity: `OBESITY_indicator`

diabetes: `DIABETESCOMPLICATED_indicator` or `DIABETESUNCOMPLICATED_indicator`

tobacco use (ever smoker before inclusion): `TOBACCOSMOKER_indicator`

data partner (study site): `data_partner_id`

source common data model format: `cdm_name`

Long COVID diagnosis: `LL_Long_COVID_diagnosis_indicator`

References

Bang, Heejung and James M Robins (2005). "Doubly robust estimation in missing data and causal inference models". In: *Biometrics* 61.4, pp. 962–973. DOI: 10.1111/j.1541-0420.2005.00377.x.

Lendle, Samuel D. et al. (2017). "ltmle: An R Package Implementing Targeted Minimum Loss-Based Estimation for Longitudinal Data". In: *Journal of Statistical Software* 81.1, pp. 1–21. DOI: 10.18637/jss.v081.i01. URL: https://www.jstatsoft.org/index.php/jss/article/view/v081i01.

Petersen, Maya et al. (2014). "Targeted Maximum Likelihood Estimation for Dynamic and Static Longitudinal Marginal Structural Working Models". In: *Journal of Causal Inference* 2.2, pp. 147–185. DOI: 10.1515/jci-2013-0007.

Robins, James (1986). "A new approach to causal inference in mortality studies with a sustained exposure period—application to control of the healthy worker survivor effect". In: *Mathematical Modelling* 7.9, pp. 1393–1512. ISSN: 0270-0255. DOI: https://doi.org/10.1016/0270-0255(86)90088-6. URL: https://www.sciencedirect.com/science/article/pii/0270025586900886.

Zhou, Andrea (July 2025). *National-Clinical-Cohort-Collaborative/Logic- Liaison-Template-N3C-Covid-Enclave-Covid-19-Cases- and-Controls-Fact-Tables: July 2025 Release*. Version v1.0. DOI: 10.5281/zenodo.15990628. URL: https://doi.org/10.5281/zenodo.15990628.
