# Supplementary material for: COVID-19 vaccination timing, relative to acute COVID-19, and subsequent risk of long COVID
Source: eBioMedicine. 2026 Jul 24;130:106382. doi: 10.1016/j.ebiom.2026.106382 (PMC13445364; doi:10.1016/j.ebiom.2026.106382)
Supplement: Supplementary file 2 [file mmc6.docx]

| **Authorship First Name Harmonized** | **Authorship Middle Name Harmonized** | **Authorship Last Name Harmonized** | **Authorship Full Name** | **Email Address** | **ORCID numeric** |
| --- | --- | --- | --- | --- | --- |
| Christopher | G | Chute | Christopher G Chute | chute@jhu.edu | 0000-0001-5437-2545 |
